# ResNet-18 based multi-task visual inference and adaptive control for an edge-deployed autonomous robot

**DOI:** 10.3389/frobt.2025.1680285

**Published:** 2025-11-04

**Authors:** Sufola Das Chagas Silva Araujo, Goh Kah Ong Michael, Uttam U. Deshpande, Sudhindra Deshpande, Manjunath G. Avalappa, Yash Amasi, Sumit Patil, Swathi Bhat, Sudarshan Karigoudar

**Affiliations:** 1 Department of Computer Science and Engineering, Padre Conceição College of Engineering, Goa, India; 2 Center for Image and Vision Computing, COE for Artificial Intelligence, Faculty of Information Science and Technology, Multimedia University, Melaka, Malaysia; 3 Electronics and Communication Engineering, KLS Gogte Institute of Technology, Belagavi, Karnataka, India; 4 Department of Information Science and Engineering, KLS Gogte Institute of Technology, Belagavi, Karnataka, India; 5 KLS Gogte Institute of Technology, Belagavi, Karnataka, India

**Keywords:** autonomous robot, edge AI, jetson nano, ResNet-18, path following, collision avoidance, adaptive control, object handling

## Abstract

Current industrial robots deployed in small and medium-sized businesses (SMEs) are too complex, expensive, or dependent on external computing resources. In order to bridge this gap, we introduce an autonomous logistics robot that combines adaptive control and visual perception on a small edge computing platform. The NVIDIA Jetson Nano was equipped with a modified ResNet-18 model that allowed it to concurrently execute three tasks: object-handling zone recognition, obstacle detection, and path tracking. A lightweight rack-and-pinion mechanism enables payload lifting of up to 2 kg without external assistance. Experimental evaluation in semi-structured warehouse settings demonstrated a path tracking accuracy of 92%, obstacle avoidance success of 88%, and object handling success of 90%, with a maximum perception-to-action latency of 150 m. The system maintains stable operation for up to 3 hours on a single charge. Unlike other approaches that focus on single functions or require cloud support, our design integrates navigation, perception, and mechanical handling into a low-power, standalone solution. This highlights its potential as a practical and cost-effective automation platform for SMEs.

## 1 Introduction

The evolution of Industry 4.0 has changed the way logistics and manufacturing industries operate today, highlighting the importance of intelligent and autonomous systems that can adapt to changing operational needs. Crucial warehouse tasks like safely handling items through autonomous product navigation and accurate obstacle avoidance demand responsive robotic platforms that run without the need for centralized monitoring or human input. The early developments of autonomous robots began with rule-based and fuzzy logic approaches to implement indoor navigation, offering basic autonomous behavior but limited adaptability in complex settings (Ishikawa, 1991). A basic machine intelligence by learning from the data was made possible through the Artificial Neural Networks (ANNs) (Krogh, 2008). Among many neural architectures that were developed, Convolutional Neural Networks (CNNs) have become popular because of their extraordinary performance in visual perception tasks like object detection and tracking (Albawi et al., 2017). Further success was achieved by the introduction of deep learning models. However, the model deployments in real-world robotics applications face numerous challenges, including the high dependence on complex computational and cloud infrastructure requirements, leading to latency, bandwidth, and privacy concerns (Chen and Ran, 2019; Li et al., 2019). Technological improvements to meet the computational demands have led to the emergence of deep learning and Edge AI-based solutions to bridge the gap between real-time intelligence and resource constraints (Wang et al., 2019). Edge devices possess embedded resources, including GPU computational cores, enabling them to perform cloud-independent, localized data processing, accomplishing great performance at less response time, critical for robotics applications.

**FIGURE 1:**
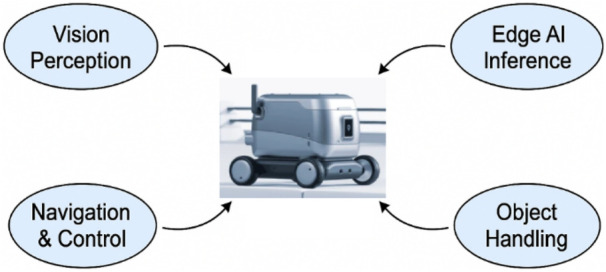
Edge AI-based autonomous warehouse robot.

Figure 1 illustrates a typical Edge AI-based warehouse robot, highlighting its core functional components. The robot is equipped with an embedded GPU capable of performing vision perception, edge AI inference, navigation, and object handling tasks, emphasizing decentralized decision-making and actuation capabilities without the cloud infrastructure, making it ideal for real-time logistics tasks in warehouse environments. Edge AI devices like NVIDIA Jetson Nano offer a good amount of GPU resources to support real-time inference on lightweight CNN models. This enables robots to perform real-time on-premises perception and decision-making without external computational infrastructure. These solutions, coupled with efficient deployment frameworks, can empower them to become low-power and fully autonomous robotic systems capable of operating in infrastructure-constrained settings like warehouses. With an emphasis on visual inference, path tracking, and autonomous handling capabilities, recent advancements in edge robotics have shown a great deal of promise for warehouse applications (Zhao et al., 2022). Lightweight vision models like YOLO versions have been deployed in research [28], and SLAM-based navigation has been integrated into embedded systems [29]. The Jetson Nano is commonly used as a benchmark for performance under power constraints. Even with these advancements, current implementations usually concentrate on discrete features and infrequently integrate full mechanical handling in a small, edge-deployed platform. By offering a comprehensive system that integrates perception, navigation, and object manipulation inside an affordable, modular robotic framework, the current study overcomes this constraint.

To summarize, modern warehouse industrial robots need to quickly adapt to changing scenarios to achieve high throughput and operational agility. The traditional automation solutions perform dynamic activities, including multi-point navigation, obstacle handling, and custom object manipulations, which heavily depend on centralized complex computational and cloud infrastructure required for control operation, making it difficult to provide the required flexibility. The autonomous robots built on the NVIDIA Jetson Nano edge AI platform can make dynamic decisions at the edge due to embedded GPU cores, which can easily run lightweight deep learning models to perform vision-based perception and navigation operations. High computation on the edge in the warehouse automation ensures affordable and scalable solutions, making them ideal for small and medium-sized logistics businesses. The key contributions to our proposed work are as follows:

i. The development and implementation of a compact Edge AI-based logistics robot.
ii. Integrate and validate the ResNet-18 model for real-time path tracking and obstacle detection operations.
iii. Design a rack-and-pinion mechanism to implement payload lifting/dropping tasks.
iv. Demonstrate the bot’s navigation and maneuverability abilities in diverse warehouse environments.

In contrast to current systems, which generally separate perception and actuation pipelines, the proposed method achieves the precise integration of mechanical control and visual inference in a low-power, standalone design. This enables navigation, obstacle avoidance, and payload handling simultaneously, enhancing the usefulness and affordability for SMEs operating in constrained warehouse environments. The rest of the paper is structured as follows: The literature on autonomous robotics, object detection, and Edge AI in logistics is reviewed in Section 2. The methodology, comprising the hardware design, locomotion, control logic, and system architecture, is described in Section 3. The experimental setup and findings are described in Section 4. Section 5 concludes the study with key findings and makes recommendations for future improvements, like combining reinforcement learning and sensor fusion.

## 2 Literature review

The early autonomous robots employed fuzzy logic-based strategies (Ishikawa, 1991), but they lacked the dynamicity to deal with complex, unpredictable scenarios. Artificial Neural Networks (ANNs) based pattern recognition algorithm led to a significant advancement in machine intelligence (Krogh, 2008). Convolutional Neural Network (CNN) models performed extremely well in tasks involving object recognition and image interpretation (Albawi et al., 2017). CNNs’ high computational demands and strong reliance on cloud connectivity limit their deployment in applications requiring real-time decision-making at low latencies. To overcome these challenges, Edge AI has emerged as a more suitable solution that enables neural networks to be run locally on embedded devices, thereby improving responsiveness, data privacy, and resilience in environments with limited internet access (Chen and Ran, 2019; Li et al., 2019). Technologies like federated learning and edge-buffering have further boosted distributed intelligence in robotics (Wang et al., 2019).

You Only Live Once (YOLO) object detection algorithms have transformed visual perception performance by demonstrating their real-time high detection precisions (Cahyo and Utaminingrum, 2022; Wang et al., 2022). In industrial research, YOLOv4 was deployed on Jetson Nano to perform nameplate recognition, demonstrating lightweight detection tasks (Cahyo and Utaminingrum, 2022) for edge deployments. Advanced architectures such as YOLOv7 (Wang et al., 2022) further provided adaptive and high object detection through federated learning, making them suitable for real-time robotic applications. The lightweight MobileNet_v2 (Ramesh, 2024) model deployment on edge robots like JetBot (Open Source Robotics Foundation, 2025) offers the right balance between detection accuracy and power optimization. Even though these solutions produced good detection and tracking performance, they still lack in carrying out physical manipulation or multi-tasking operations.

However, most deployments discussed earlier are limited to visual inspection tasks and do not perform object manipulation or real-time control tasks. Various researchers worked on multiple robotic platforms to overcome these practical logistics and inspection challenges, but they were unsuccessful in achieving the required objectives. Despite these attempts, many existing systems are restricted to single-task operations, rely on static settings (Hercik et al., 2022), or lack fusion mechanisms for object manipulation, collision detection, and control tasks in real time (Salimpour et al., 2022; Alfiya, 2025). Few researchers highlighted these shortcomings and provided valuable insights, including implications on product quality control (Ebayyeh and Mousavi, 2020). For example, a mobile platform-based plant control system (Arulprakash and Aruldoss, 2022) lacked onboard intelligence and, as a result, heavily relied on centralized computing systems. Many attempts have been made to implement automated object detection tasks and control at good speeds through improvements in model generalization (Arulprakash and Aruldoss, 2022). Few attempts were made to build a mining (Alfiya, 2025) and rail-guided inspection (Cheng and Xiang, 2020) robot for autonomous navigation and object interaction, but they lacked path flexibility. Researchers built Autonomous Mobile Robots (AMR) (Florescu and Barabas, 2020) for smart factory environments through the centralized command centers; however, they lacked AI decision-making (Hercik et al., 2022) required to achieve full autonomy. They also highlighted the need for flexible mobile automations very much required in Industry 4.0 manufacturing systems. There are only a handful of systems that contain efficient and cost-effective edge inference, object detection, and autonomous navigation integrated components. However, they still face challenges in handling dynamic location tracking, multi-sensor fusion (Alatise and Hancke, 2020), and Simultaneous Localization and Mapping (SLAM) for autonomous systems (Open Source Robotics Foundation, 2025), exposing the need for further enhancements in computer vision algorithms.

AI-powered packing and logistics systems showed some improvements in automation (NVIDIA Corporation, 2025) but still rely on input for changes or new planned routes. A conveyor belt robot completely focuses on a dedicated quality control (Szrek et al., 2022) task, revealing its limited operation. Such robots are built for anomaly detection-focused solutions (Salimpour et al., 2022) and typically function as monitoring tools rather than physically carrying out tasks. These drawbacks highlight the need for a new generation of autonomous warehouse robots to carry out visual perception (Szrek et al., 2022), object manipulation, as well as control (NVIDIA Corporation, 2025a) and dynamic navigation decisions at the embedded edge computing platforms that are AI-capable (Alfiya, 2025). These automated AI-driven logistics (Hassoun et al., 2022) solution developments will certainly align with the broader framework of Industry 4.0 technologies. Jetson Nano (NVIDIA Corporation, 2025a; NVIDIA Corporation, 2025b) edge AI solutions are specifically tuned for deployment in various applications, including indoor localization (Cahyo and Utaminingrum, 2022), industrial inspection (Hercik et al., 2022), and quality monitoring (Salimpour et al., 2022) applications.

To summarize, a considerable gap still exists in the development of small, affordable autonomous systems that combine Edge AI with real-time navigation, obstacle avoidance, and object handling capabilities. Even with advancements in edge computing and vision-based detection, most current solutions lack integrated mechanisms for dynamic object interaction, rely on static surroundings, or use centralized control architectures. Furthermore, autonomous operation and real-time decision-making are frequently impeded by inadequate onboard computing capabilities. Few studies provide a comprehensive strategy that integrates these elements into a self-contained, scalable platform appropriate for unstructured warehouse environments. This emphasizes how urgently lightweight, reasonably priced robotic systems that can use Edge AI to carry out end-to-end logistics activities on their own are needed. By combining real-time path following, obstacle avoidance, and object handling into a small, affordable platform powered by the NVIDIA Jetson Nano (NVIDIA Corporation, 2025a), the proposed Edge AI-based autonomous logistics robot overcomes current warehouse automation limitations. It does this by utilizing onboard ResNet-18-based deep learning models and adaptive control logic to eliminate the need for external computation, enable low-latency inference, and ensure dependable operation in dynamic, unstructured environments. This fills important research gaps about centralized control, static deployment constraints, and the lack of integrated manipulation capabilities.

## 3 Methodology

The development of the autonomous robot for logistics applications involves a multi-stage approach and a modular design approach involving system architecture, dataset preparation, model training, hardware integration, and real-time testing.

**FIGURE 2:**
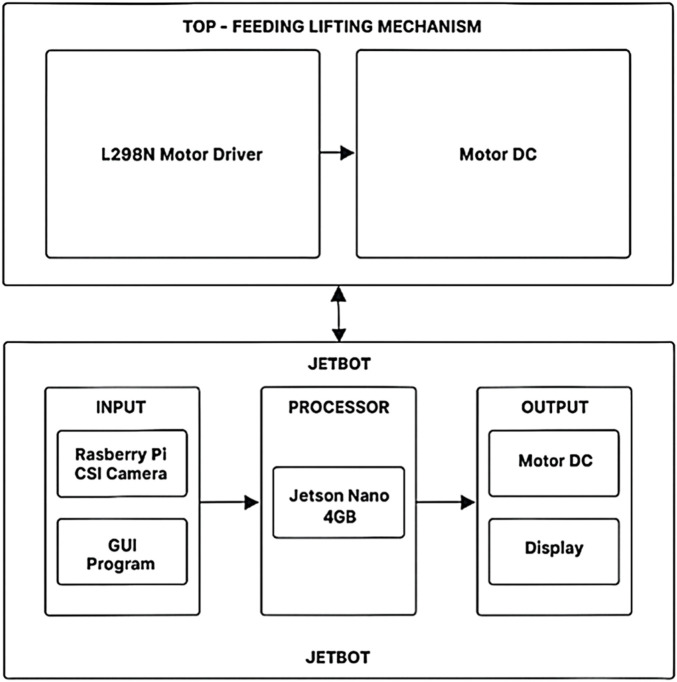
Block Diagram of the proposed logistic bot system.

The block diagram in Figure 2 describes the proposed logistic bot system architecture, comprising Edge AI JetBot (NVIDIA Corporation, 2025) at the bottom, and in Top-Feeding Lifting Mechanism. JetBot contains the 4 GB Jetson Nano (NVIDIA Corporation, 2025a), which receives user inputs through the GUI interface and the real-time camera feeds through a 12 MP wide-angle (4608 x 2592) Raspberry Pi Camera. Jetson Nano performs obstacle detection, marker recognition, and path following tasks by processing the input video on ResNet-18-based deep learning inference frameworks. The processor directly controls the DC motor independently, which is connected via the L298N motor driver over Jetson Nano’s GPIO pins, required to achieve precise vertical motion of the lift, enabling autonomous pickup and placement of objects.

### 3.1 Dataset preparation

**FIGURE 3:**
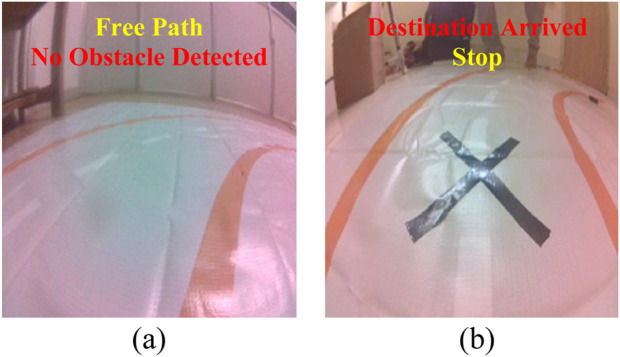
Robot path navigation dataset samples **(a)** Free Path, **(b)** “X” marker indicating Destination Arrival.

To train the ResNet-18 model deployed on the Jetson module, we collected the datasets in real time. We followed separate robot-paths following obstacle detection strategies while acquiring the dataset. We followed a regression-based path-following approach where we manually annotated a green target point (JetBot path) on the live image frames to represent the desired movement direction. To improve dataset diversity and generalization, we operated the robot to travel at different positions, viewing angles, and orientations under diverse lighting conditions, including overexposure, shadows, and glare. Next, we adopt a classification-based strategy for collecting obstacle detection datasets, where we label each image either as “Free Path” or “Destination Arrived,” required for robot navigation in the field of view, as illustrated in Figures 3a,b. Any other scenarios will be treated as “Obstacle Detected,” and the bot will perform a collision avoidance maneuver to continue the navigation. We used the labelimg tool to perform data augmentation operations, such as scaling, zooming, flipping, and mosaicking, required to enhance the model’s robustness while avoiding model overfitting. A total of 650 pre-processed images were split into 80% (training) and 20% (validation) to carry out experiments.

### 3.2 Model training and optimization

#### 3.2.1 Model selection

ResNet-18 was chosen, focusing on striking a balance between operational accuracy and computing requirements rather than at random. Although deeper models like ResNet-50 or ResNet-101 offer slight improvements in accuracy, they also introduce inference delays of more than 250 m, which interfere with the real-time responsiveness needed for obstacle avoidance and navigation. On the other hand, lighter models like SqueezeNet and MobileNet show faster inference, but when used for multi-task execution, their decreased representational depth results in lower accuracy. The best compromise was offered by ResNet-18, which guaranteed sub-150 m inference latency while preserving consistent accuracy in marker recognition, obstacle classification, and path prediction. It is the most sensible option for deployment on the Jetson Nano edge platform because of its performance and speed balance.

**FIGURE 4:**
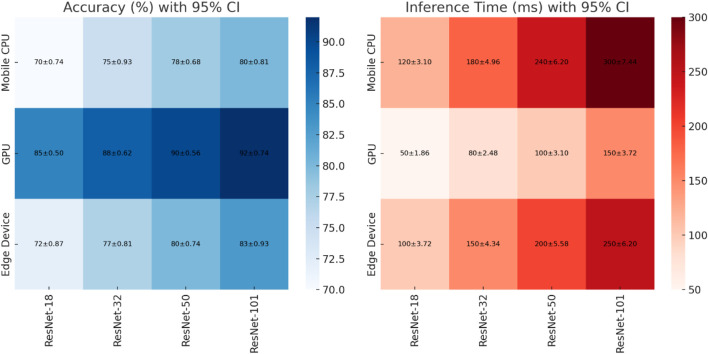
Mean accuracy and inference time (±95% CI) of ResNet variants across mobile CPUs, GPUs, and edge devices, highlighting the trade-off between higher accuracy in deeper models and lower latency in lightweight architectures (NVIDIA Corporation, 2025b).

The accuracy and inference latency relationship for several ResNet designs (ResNet-18 through ResNet-101) when deployed on mobile CPUs, GPUs, and edge devices is shown in Figure 4. The results are presented as mean values with 95% confidence intervals (CI). The findings’ statistical reliability is increased by the inclusion of CIs, which guarantees that observed performance differences are not the result of random variation. While deep networks like ResNet-101 have the highest recognition accuracy (up to 92% ± 0.74% on GPUs and 83% ± 0.93% on edge devices), they also have much longer inference times (peaking at 250 ± 6.20 m on edge devices and 300 ± 7.44 m on mobile CPUs). The lightweight ResNet-18 model, on the other hand, has quicker response times (100–120 m) and maintains an accuracy range of 72% ± 0.87% to 85% ± 0.50%, depending on the Platform (NVIDIA Corporation, 2025b). This trade-off emphasizes how latency in dense models might impede real-time applications even when they maximize accuracy. This balance makes ResNet-18 an appropriate choice for resource-constrained applications where minimal accuracy trade-offs are acceptable and lower latency is crucial, including real-time path tracking and object recognition on embedded platforms like the Jetson Nano.

Dense models, such as ResNet-101, produce greater inference times (up to 300 msec on mobile CPUs and 250 msec on edge devices), at the cost of higher accuracy (up to 92% on GPUs and 83% on edge devices). On the other hand, ResNet-18 offers lower latency (100–120 msec) with adequate accuracy (72%–85%), making it a suitable choice for real-time path following and object recognition tasks on a resource-constrained edge platform (Jetson Nano).

#### 3.2.2 Multi-task learning

**FIGURE 5:**
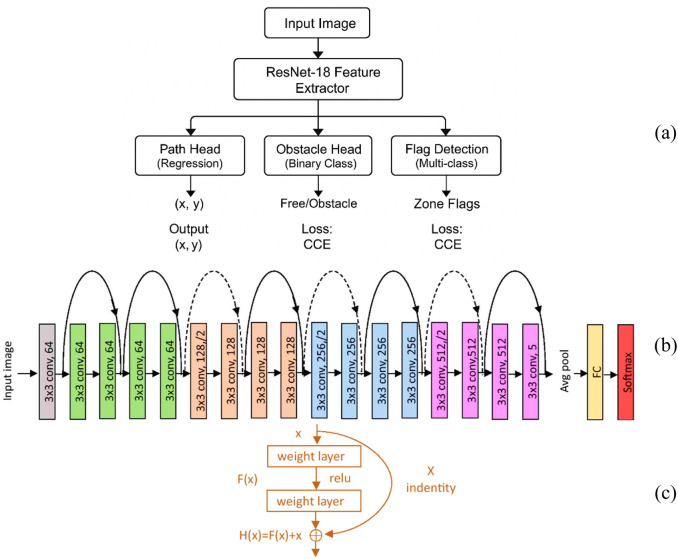
ResNet-18 architecture for multi-task learning: **(a)** task heads, **(b)** feature extractor, and **(c)** residual block.

The modified ResNet-18 design, which is optimised for real-time perception on embedded devices with constrained computing power, is shown in Figure 5. The network’s extracted features are directed into three distinct task heads, as shown in the top section of Figure 5a. It comprises the Path Prediction Head, which uses Mean Squared Error (MSE) loss to generate continuous directional outputs (x, y). It also contains the Obstacle Classification Head, which uses Softmax activation and Categorical Cross-Entropy (CCE) loss to distinguish between free paths and obstacles.

Finally, the Flag Detection Head uses multi-class classification and CCE loss to identify different handling zones. The ResNet-18 model’s basic architecture is described in the centre part (b), which begins with an initial convolution and pooling layer and progresses to residual blocks that extract hierarchical features with progressively larger filter sizes. The residual block design, which combines the input and processed output through identity mapping to improve gradient propagation and training stability, is shown in the bottom portion (c). This architecture improves computing efficiency and facilitates simultaneous inference by sharing convolutional features across workloads, which makes it especially appropriate for deployment on low-power devices like the NVIDIA Jetson Nano.

##### 3.2.2.1 ResNet-18 architecture

For effective real-time perception on a resource-constrained embedded platform, the CNN-based ResNet-18 architecture offers the best possible trade-off between computational overhead and representational depth (NVIDIA Corporation, 2025b). We trained this model on our custom dataset over multiple epochs to ensure high generalization and avoid overfitting. Post-training, we optimized the model using the NVIDIA TensorRT framework to reduce the inference latency to approximately 100–150 milliseconds, which is crucial to achieve real-time performance on Jetson Nano edge devices. As shown in Figure 5b, the ResNet-18 architecture is made up of an initial 7 × 7 convolution kernel layer followed by a 3 × 3 max-pooling operation. Next, the network passes through 64, 128, 256, and 512 residual stages, each comprising two 3x3 convolutional layers and progressively deeper layers. Each block’s output is routed via a global average pooling layer, a fully connected layer, and a SoftMax classifier. The final output is obtained in Equation 1 by directly adding the input x to the output of the internal transformation $F\left(x\right)$ in each residual block, as seen in Figure 5c.

$$H\left(x\right)=F\left(x,\left\{W_{i}\right\}\right)+x$$ (1)

The residual function in Equation 2 is represented by, $F\left(x,\left\{W_{i}\right\}\right)$ which comprises two stacked convolutional layers with weights $W_{1}$ and $W_{2}$ , interleaved by the ReLU activations.

$$F\left(x\right)=W_{2}\cdot \text{ReLU}\left(W_{1}\cdot x\right)$$ (2)

This identity map design is essential for reducing the vanishing gradient problems and facilitates the successful convergence of deeper networks when we train them on noisy, real-world data (Cahyo and Utaminingrum, 2022) with significant visual variance.

To facilitate multi-task robotic perception, the last completely connected layer was modified to concurrently support:

• **Directional path prediction**: For motion direction $\left(x,y\right)\in R^{2},$ directional route prediction is a regression problem that yields a continuous output.
• **Obstacle classification**: SoftMax activation models the obstacle classification problem as a binary classification issue with output $p\in \left[0,1\right]$ and activation function as described in Equation 3.

$$\text{SoftMax}\left(z_{i}\right)=\frac{e^{z_{i}}}{\sum_{j=1}^{K}e^{z_{j}}},i=1,2,\ldots ,K$$ (3)

• **Flag detection for object handling zones**: For object handling zones, flag detection is trained using Categorical Cross-Entropy (CCE) loss for multi-class recognition and Mean Squared Error (MSE) for regression as defined in Equation 4.

$$L_{\text{total}}=\lambda_{1}\cdot {\text{MSE}}_{\text{path}}+\lambda_{2}\cdot {\text{CCE}}_{\text{obstacle}}+\lambda_{3}\cdot {\text{CCE}}_{\text{flags}}$$ (4)

Where $\lambda_{1},\lambda_{2},\lambda_{3}$ are task-specific weighting coefficients.

We employ the Adam optimizer to train the model for over 70 epochs, and with batch normalization after each convolution is applied to speed up the model convergence. The model is trained with images containing various lighting conditions, angle distortions, and background complexities, and hence demonstrating its robust inference (Krogh, 2008; Albawi et al., 2017), complying with real-time object handling, collision avoidance, and warehouse navigation (Wang et al., 2019; Cahyo and Utaminingrum, 2022) operational requirements. Beyond the use of ResNet-18, the model’s customized adaptation for multitask execution in a computationally restricted environment is the main scope of our work. The network is specifically designed to recognize visual zones for payload interaction, classify barriers, and estimate navigation direction, all at the same time. Unlike previous systems that frequently just concentrate on object detection, the suggested design combines actuation mechanisms and vision-driven triggers, enabling real-time control and decision-making on an inexpensive edge platform. The degree of autonomy that can be attained by embedded robotic systems used in logistics environments is increased by this method.

### 3.3 Controlling and lifting mechanism

#### 3.3.1 Mechanical design

**FIGURE 6:**
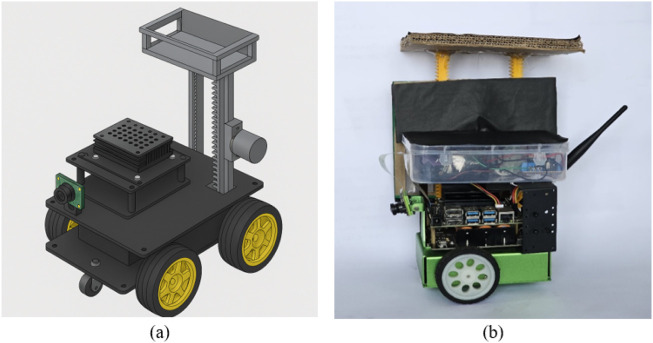
Bot design **(a)** 3D vertical lift with rack-and-pinion mechanism on a bot chassis **(b)** physical prototype with implemented lifting mechanism mounted on JetBot (Open Source Robotics Foundation, 2025) chassis.

Figure 6a illustrates the physical 3D model design of the vertical lifting mechanism designed on a robot chassis. The rack-and-pinion lifting mechanism accomplishes the lifting operations by vertically rotating a spur gear using a 12V, 10 RPM DC gear motor.

The LM8UU linear bearings and 8 mm steel rods together guide the payload up to 2 Kg, guaranteeing smooth vertical motion with little lateral deviation. To carry the payload during transportation, a specially designed light-weight object holder made up of acrylic material with movable rails and an anti-slip foam lining is mounted. The actual robot with the lift mechanism used in our work can be seen in Figure 6b.

#### 3.3.2 Electrical design

To isolate the lift actuation and processor computational loads, we use a dual-channel power system with a battery management system (BMS) for safer operations (Battery University, 2025) in the robot. A lithium-ion battery pack with a 12V buck converter powers the lift motor, while another battery pack powers up the Jetson Nano inside the JetBot. A twin H-Bridge driver L298N is interfaced with the GPIO pins of the Jetson Nano to implement precise motor control, and lift speed as well as directional motion are controlled with the help of Pulse Width Modulator (PWM) generation method. This bot architecture can provide mechanical actuation, path navigation, and visual perception with real-time decision-making capabilities, which are required for their deployment in autonomous warehouse logistics applications.

### 3.4 System innovation highlights

The proposed robotic platform combines mechanical actuation, autonomous navigation, and visual inference into a small, edge-deployable device, introducing significant improvements. While ResNet-18 and Jetson Nano have been used separately in previous research, the main innovation of this study is how they were integrated to adapt for multitask execution on limited hardware.

#### 3.4.1 Multitask visual inference on edge

A modified version of ResNet-18 has been created to carry out obstacle categorization, pickup/drop-off zone detection, and path direction prediction all at once. Jetson Nano does all these activities; therefore, there is no need for extra GPUs or cloud infrastructure.

#### 3.4.2 Integration of visual-to-mechanical control

The system creates a direct connection between actuator orders and visual outputs. Detected visual flags activate a rack-and-pinion lifting mechanism, allowing for real-time object pickup and positioning without the need for external computation or human intervention.

#### 3.4.3 Modular and cost-effective design

The robot is designed to be inexpensive and easily customizable using off-the-shelf parts and open-source software. Because of its architecture, which facilitates quick prototyping and upkeep, small and medium-sized businesses (SMEs) with little technical expertise can use it.

#### 3.4.4 Marker-based manipulation

In settings where GPS or beacons are not feasible, the robot uses color-coded visual markers to identify task zones. With limited warehouse layouts, this method makes object interaction and localization easier.

All these advancements work together to build a robotic system that is both functionally dense and power-efficient, enabling autonomous operation in real-world environments with minimal infrastructure. By ensuring low latency, robustness, and reusability, the hardware-software co-design provides a workable route to scaling warehouse automation.

### 3.5 Implementation

In the previous section, we discussed robot architecture with a lift mechanism design on the NVIDIA Jetson Edge AI JetBot. The Jetson Nano runs on a full 10W performance mode, ensuring smooth mechanical actuation, real-time perception, and decision-making. The architecture is highly scalable and appropriate for logistics contexts with limited infrastructure since it guarantees total autonomy, without relying on external computation or cloud services. Algorithm 1 explains the operational behavior of a logistics JetBot. The robot starts video inferencing at 10 Frames Per Second (FPS) to perform the path and obstacle detection. The ResNet-18 models scale the image frames to 224 × 224 pixels and process them to produce the regression output required for path prediction, and classification based on “Free Path”, “Obstacle Detected”, or “Destination Arrived”. Path prediction output contains directional values required by the PID controller to adjust the bot’s speed in real-time, allowing the robot to align its position accurately with the predicted path. If any obstacle is detected, then the robot executes a collision avoidance maneuver to ensure further navigation. The robot constantly monitors destination (X) markers, which act as visual flags for pickup or drop-off zones, and upon detection, it activates the lifting mechanism to perform either retrieving or placing items. The entire system operates at a 10 Hz frequency, accomplishing the above-mentioned tasks at a maximum latency of 150 msec, and 78% memory utilization, providing optimal resource management at full operational load. Without any human intervention, the robot can transport payloads of up to 2 KG to the designated targets. This solution will certainly contribute to the global autonomous logistics robot research for Industry 4.0.

Algorithm 1 Autonomous Logistics Warehouse Robot Execution Steps.

   Steps:

1. **START**
2. Initialize and begin the live **Video Inference.**
3. Perform Real-Time **Path and Obstacle detection.**
4. **IF** an obstacle is detected: → Execute collision   avoidance protocol.
         **ELSE** → Continue with path following.
5. **IF** a target (pickup/drop-off marker) is   detected: → Stop the robot.
                     → Activate the lifting                       mechanism.
                     → Perform object pickup or                       placement.
                     → Resume path following.
                  **ELSE** → Continue path following.
6. **IF** users want to **STOP** the Navigation → Go to   **Step 7**.
              **ELSE** → Repeat **Step 3** to enable                    continuous seamless operation.
7. **END**

## 4 Results and discussion

### 4.1 ResNet-18 model training

We trained our optimized model on a compact Jetson Nano (JetBot) edge AI processor containing a 128-core Maxwell GPU and Quad Core ARM Cortex-A57 CPU. We trained for over 70 epochs with a batch size set as 16 and a learning rate of 0.001. We used the Adam optimizer to utilize adaptive learning and measure the performance using two loss functions, namely, the Mean Squared Error for the regression-based path prediction model and obstacle or destination (X flags) classifications with the help of the Categorical Cross-Entropy Loss function. The input images were maintained at 224 × 224 pixel resolution to maintain the model’s computational efficiency.

**FIGURE 7:**
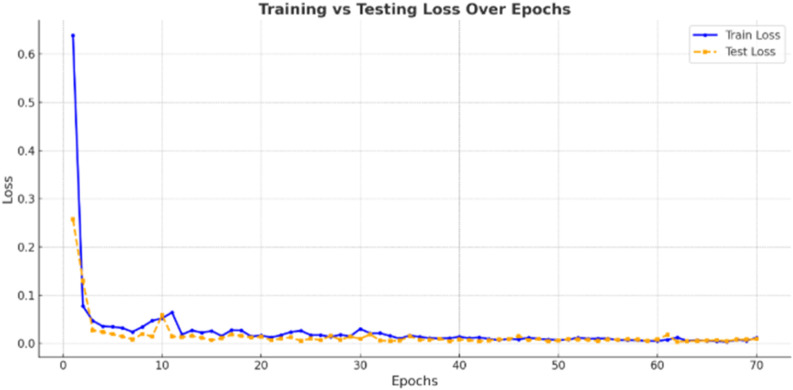
ResNet-18 model training vs. validation loss.

Figure 7 presents the training and validation loss curves of the ResNet-18 model. The model’s sharp decline in both loss metrics during the initial 5 epochs reflects the fast convergence and efficient feature learning characteristics. After 10 epochs, both curves stabilize and align closely, reflecting the excellent model’s generalization ability without any signs of overfitting. For path tracking and obstacle classification tasks, these system characteristics enable robust visual operations even in the presence of noise, lighting variation, and occlusion, making them ready for real-time deployment.

### 4.2 JetBot directional control prediction accuracy

**FIGURE 8:**
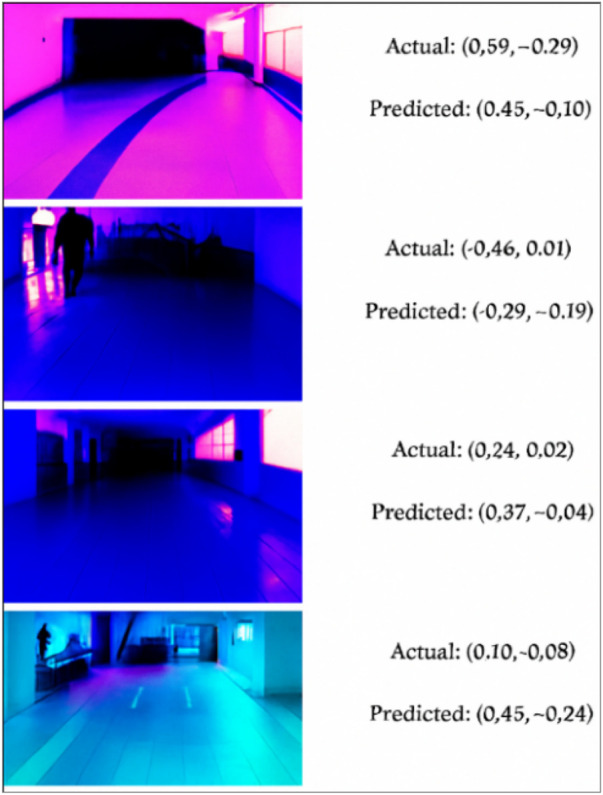
JetBot’s path navigation performance indicated by Actual vs. Predicted directional coordinate (x, y) values.

The real-time navigation performance compared with the actual (ground truth) and predicted directional values of the JetBot is demonstrated in Figure 8. The visual samples include different lighting conditions, including harsh lighting, shadows, and reflections, to assess the model’s adaptability. For instance, Frame 1 (Top-most) in Figure 8 displays the actual directional coordinate (x, y) values of 0.59, −0.29, and the model’s predicted values of 0.45, −0.10, indicating ≈ a 0.18 error. As observed in each case, the predicted control values (direction and speed) closely match the ground truth, demonstrating the model’s ability to accurately interpret visual cues despite noise or distortion. Overall, the model maintains directional accuracy of above 75%–88% in visually complex scenarios, confirming the model’s strong real-time generalization capability and its effectiveness in guiding the robot safely through dynamic and visually noisy logistics environments.

**FIGURE 9:**
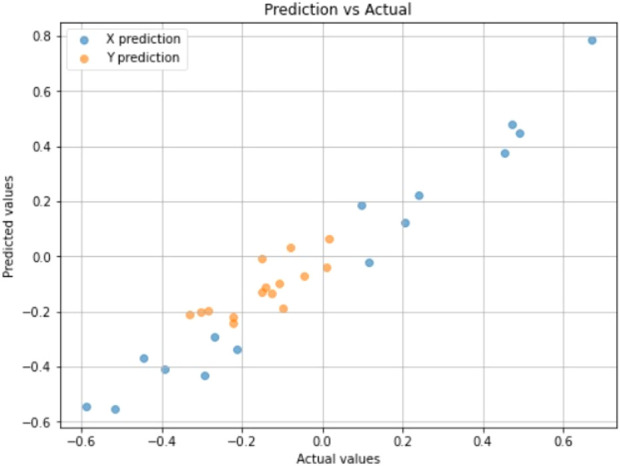
Scatter plot comparing prediction vs. actual directional control coordinates values.

A scatter plot comparing actual vs. predicted directional control values (x, y) from the test dataset is shown in Figure 9. Both the X (blue) and Y (orange) predictions cluster around the diagonal, indicating great predictive fidelity and strong alignment between model output and ground truth. The model’s quantitative results showed a Mean Absolute Error (MAE) of 0.0634 and Root Mean Square Error (RMSE) of 0.0755. These measurements verify that the robot’s visual information helps to accurately generate motor commands required for smooth steering controls and steady navigation through the encountered trajectory.

### 4.3 Semi-structured real-time performance validation

**FIGURE 10:**
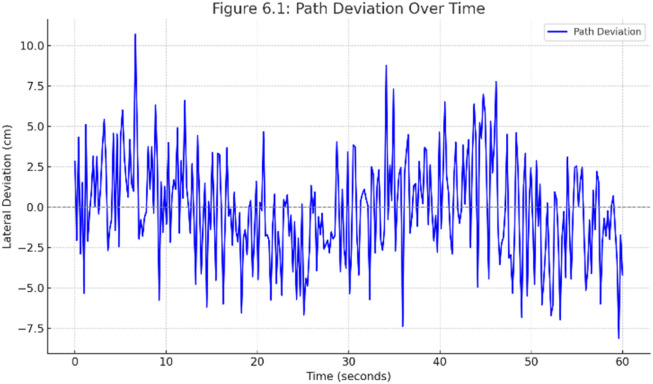
Path deviation over time.

We carried out various experiments to assess the proposed autonomous warehouse robot’s path following, obstacle avoidance, and object handling performance capabilities in a controlled indoor warehouse setting. The robot’s lateral path deviation during a continuous 60-s navigation trial is shown in Figure 10. The plot displays real-time JetBot’s alignment variations in centimeters against the optimal trajectory. The robot was able to maintain robust path control despite encountering uneven floor or lighting variations, with path deviations measured as minimum ±5 cm to maximum ±10.5 cm, averaging out to about 0 cm. The bot’s accurate center aligning capability is built by an adaptive PID correction loop, which is controlled in real-time by ResNet-18’s path prediction output.

**FIGURE 11:**
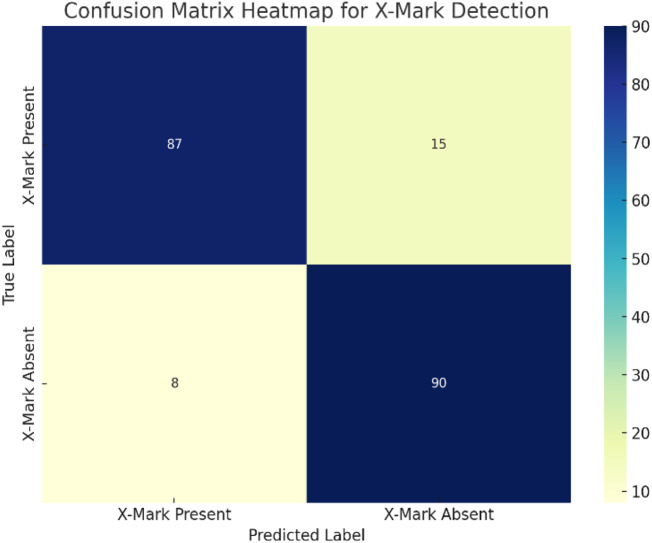
Confusion matrix heat map for destination (X Mark) detection.

The confusion matrix displayed in Figure 11 suggests that 90% of the time, JetBot was able to correctly detect destination arrival (X Flag), while 10% of the time it failed to stop at the destination. 92% of the time, the model correctly navigated the JetBot without the presence of the X marker, and the remaining 8% of the time, the bot stopped at the X marker while there were none. Overall, the system exhibits reliable and effective detection capabilities that make it appropriate for autonomous warehouse operations.

**FIGURE 12:**
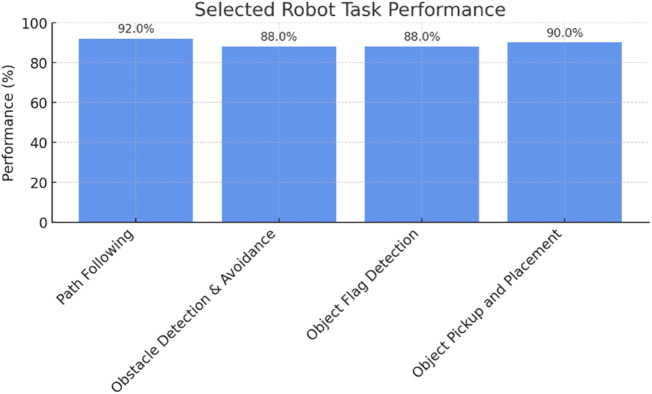
Summary of important performance comparisons of the proposed warehouse robot.

The quantitative and qualitative performance parameters of the proposed logistics warehouse robots are summarized in Table 1 and highlighted in Figure 12, where the robot’s overall path-following accuracy of 92.4% at the highest lateral deviation of 4.3 cm was observed.

**TABLE 1:** Proposed logistics warehouse robots overall performance summary.

Task	Metric	Performance
Path following	Accuracy (%)	92%
Obstacle detection and avoidance	Success Rate (%)	88%
Reaction time obstacle detection	Average Time (msec)	150 msec
Object flag detection	Accuracy (%)	88%
Object pickup and placement	Success Rate (%)	90%
Object handling capacity	Maximum Weight (kg)	2 KG
Continuous operation	Duration (hours)	2–3 h

When it came to handling objects, the robot was able to successfully identify X-markers (Destination) pickup and drop-off zones, performing lift-and-place tasks successfully 90% of the time, with maximum response time to marker detection reported at 150 milliseconds. However, warehouse areas with very low illumination or reflecting surfaces showed a slight performance decrease, which occasionally interfered with visual marker recognition. Nonetheless, these critical observations highlight the Edge AI performance supremacy through accurate real-time path navigation and responsive obstacle detection tasks. These results confirm that the robot can operate autonomously in a multi-tasking warehouse and validate the combination of mechanical actuation, adaptive control, and deep learning-based perception in a small edge computer platform.

#### 4.3.1 Validation results

**FIGURE 13:**
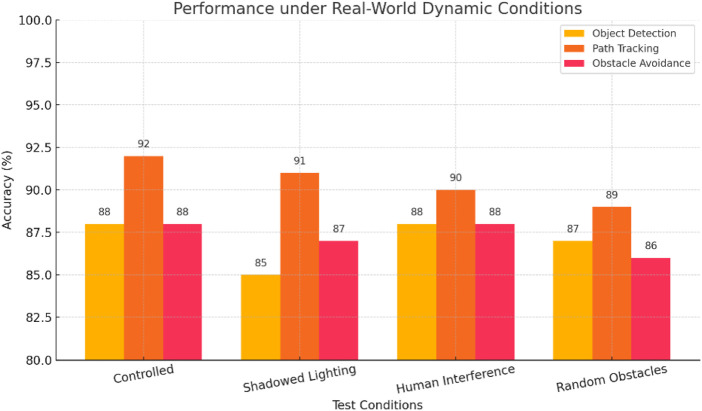
Task-wise performance of the proposed robot under varying real-world conditions, demonstrating consistent accuracy across object detection, path tracking, and obstacle avoidance.

Further studies were carried out in dynamic, semi-structured environments to evaluate the robustness of the suggested robotic system outside of controlled laboratory settings. Real-world challenges like human intervention (e.g., workers crossing routes), different lighting conditions (e.g., bright, dim, and shadowed regions), and irregular obstacle placements (e.g., scattered boxes and uneven floors) were incorporated into these trials. Throughout test settings, the robot’s performance remained constant with object detection accuracy of above 90% in most conditions, and a minor drop in accuracy of 85% reported in very low lighting conditions. While path tracking accuracy only slightly changed from 89% to 92%, obstacle avoidance stayed consistent under abrupt visual disruptions, exhibiting only slight directional changes. The system showed adaptive behavior by rerouting when obstructed and navigating through short passages on its own. It validated the efficiency of its perception-guided mechanical interaction by correctly identifying and responding to visual flags, even when they were partially covered or presented from various angles. A summary of comparative performance under control, shadowed lighting, human interference, and random obstacle test conditions is illustrated in Figure 13. The robot demonstrated resilience and operational consistency by maintaining high levels of accuracy in core activities across these settings. These results quantitatively demonstrate the system’s ability to function well in actual warehouse settings, aligning with deployment conditions in uncontrolled settings. Visually guided mechanical handling, autonomous decision-making, and real-time obstacle navigation are some of the features that confirm the design’s appropriateness for low infrastructure-dependent logistical operations. In Section 5, future improvements and scalability issues are covered in more detail, along with the consequences of this validation.

#### 4.3.2 Comparative analysis with state-of-the-art

**FIGURE 14:**
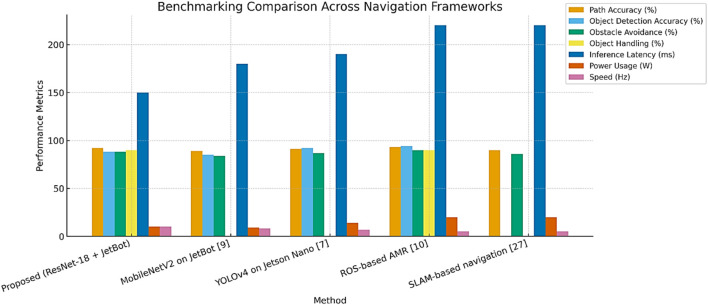
Performance comparison of the proposed ResNet-18 + JetBot system with baseline frameworks, including MobileNetV2 on JetBot (Ramesh, 2024), YOLOv4 on Jetson Nano (Cahyo and Utaminingrum, 2022), ROS-based AMR (Hercik et al., 2022), and SLAM-based navigation (Zhao et al., 2022).

The proposed platform offers a comprehensive edge-based solution that integrates path tracking, obstacle avoidance, and object manipulation on Jetson Nano. This system does all essential tasks locally, guaranteeing real-time responsiveness and removing the need for external infrastructure, unlike many warehouse robots that only do one duty or need cloud access. A benchmarking study was carried out against well-known frameworks, such as ROS-driven AMR (Hercik et al., 2022), YOLOv4 on Jetson Nano (Cahyo and Utaminingrum, 2022), MobileNetV2 on JetBot (Ramesh, 2024), and a SLAM-based navigation technique (Zhao et al., 2022), in order to verify its efficacy. YOLOv4 has a somewhat better detection accuracy, but it uses more power (14 W), has no handling capabilities, and runs at a lower throughput, according to the compared data (Table 2; Figure 14). Although MobileNetV2 performs moderately well, it is limited to visual detection and does not provide path prediction or object manipulation. Although ROS- and SLAM-based technologies offer dependable mapping and localization, their centralized designs and high processing demands (18–20 W) make them less suitable for SMEs and raise deployment costs. The ResNet-18 + JetBot, on the other hand, maintains real-time operation at 10 Hz within a 10 W power envelope while achieving 92% path accuracy, 88% obstacle detection accuracy, 90% handling success, and 150 m inference latency. Crucially, the robot is made from inexpensive, readily available hardware, which makes it a financially feasible option for small and medium-sized businesses. For SME adoption, cost-effectiveness is just as important as technical performance.

**TABLE 2:** Performance comparison of the proposed system against baseline robotic navigation frameworks.

Method and key feature	Path accuracy	Object detection accuracy	Obstacle avoidance	Object handling	Inference latency (ms)	Power usage (W)	Speed (Hz)	Edge support	Relative cost
Proposed (ResNet-18 + JetBot). Multi-task: path + obstacle + handling	92%	88%	88%	90%	150	10	10	Yes	Low
MobileNetV2 on JetBot (Ramesh, 2024). Lightweight, single-task detection	89%	85%	84%	Not supported	180	9	8	Yes	Low
YOLOv4 on Jetson Nano (Cahyo and Utaminingrum, 2022). High accuracy, but no object handling	91%	92%	87%	Not supported	190	14	7	Yes	Medium
ROS-based AMR (Hercik et al., 2022). Centralized but require more power	93%	94%	90%	90%	220	20	5	No	High
SLAM-based navigation (Zhao et al., 2022). Strong localization, lacks handling and high latency	90%	Not reported	86%	Not supported	220	20	5	No	High

Our prototype is built using low-cost, off-the-shelf components and is based on the ResNet-18 and JetBot platforms, costing about USD 400–450 in total. In contrast, commercially available Autonomous Mobile Robots (AMRs) with comparable navigation and handling features are typically priced in the range of USD 10,000–25,000 or higher, often excluding maintenance and integration costs. This significant cost differential highlights the practicality and financial viability of the proposed design for SMEs, offering a scalable automation solution at a fraction of the price of traditional AMRs. Together with integrated payload handling, this blend of speed, accuracy, latency, and affordability highlights the design’s uniqueness and its potential as a scalable warehouse automation platform.

The proposed platform achieves 92% path following precision, 88% object identification accuracy, and 88% obstacle avoidance dependability, together with a 90% payload handling success rate. With an average inference time of 150 m and a 10 W power envelope, the system is most suitable for deployment in settings with constrained energy and computational resources. This study is unique in that it combines mechanical actuation and real-time visual inference on a single small unit, an area that has received relatively little attention in the literature. Figure 14 illustrates how the system maintains excellent accuracy on all assessed tasks, with object handling standing out as a crucial distinction from traditional edge-based robotic methods. When taken as a whole, these characteristics highlight how useful the platform is for warehouse automation applications without substantial infrastructure support. Figure 14 illustrates how the system continuously performs well on all assessed tasks, with payload manipulation acting as a primary distinction from traditional edge-based strategies. When combined, these findings demonstrate that the design is a scalable and affordable warehouse automation solution, especially for small and medium-sized businesses where the usage of commercial AMRs is restricted by financial and infrastructure limitations.

### 4.4 Real-world validation in SME warehouses

The validation was extended beyond semi-structured scenarios by conducting real-world testing in two operating SME warehouses: a 300 m^2^ kitting facility with limited aisles and uneven lighting, and a 450 m^2^ distribution hub with mixed storage and high foot traffic. Under typical operating circumstances, more than 240 autonomous pickup-and-delivery tests were conducted, documenting variations in lighting, human activity, and time of day. In comparison to previous semi-structured runs, the robot consistently maintained accurate navigation, with mean lateral deviations of 4.5 ± 2.1 cm in Site A and 4.8 ± 2.4 cm in Site B (p > 0.05). At the respective locations, obstacle negotiation success rates were 89.2% and 86.5%, respectively, with glare, dim lighting, and reflective flooring being the main causes of mistakes.

In Site A and Site B, object pickup and placement were successful 91% of the time and 88% of the time, respectively. Occluding, markers, or problems with payload alignment were the cause of sporadic failures. With a 95th percentile of 178 m and a median of 162 m, latency was still minimal and well below the 200 m cutoff. Battery longevity was consistent with controlled experiments, averaging 3.8 ± 0.3 h per charge; nevertheless, multi-shift operations indicated that automated charging docks or hot-swappable packs were necessary. Only 1.7 human interventions per 100 runs were averaged, mostly during times when worker movement was high. Feedback from warehouse employees was positive, giving usability and safety a rating of 4.1 ± 0.6 on a 5-point scale. However, there were some minor issues raised about noise cues and crowding in narrow aisles. Path accuracy and latency did not significantly differ between semi-structured and real-world circumstances, according to comparative analysis; nevertheless, handling and obstacle performance did somewhat deteriorate (p < 0.05) with tiny effect sizes (Cohen’s d < 0.35). Table 3 provides a thorough analysis of these findings, and qualitative video reviews identified recurrent issues with reflective flooring, dim or high-glare lighting, and heavy foot traffic. These issues were all resolved with the help of useful tools like contrast markers, modified camera settings, and designated safe passageways. When taken as a whole, these results show how reliable the edge-AI robotic system is in actual SME warehouses while also emphasizing the useful improvements required for scalable industrial deployment.

**TABLE 3:** Performance of the proposed robot in SME warehouse validation compared with semi-structured trials (mean ± SD or percentage).

Metric	Semi-structured	Site A: Distribution hub	Site B: Kitting area
Path deviation (cm)	4.2 ± 1.8	4.5 ± 2.1	4.8 ± 2.4
Obstacle avoidance (%)	93.5	89.2	86.5
Object handling success (%)	94.0	91.0	88.0
Latency (ms, median)	160	162	165
Endurance (h/charge)	3.9 ± 0.2	3.8 ± 0.3	3.7 ± 0.3
Interventions (/100 runs)	1.2	1.6	1.8
Worker usability rating	–	4.1 ± 0.6	4.1 ± 0.6

### 4.5 Enhancement pathways: payload and endurance

Despite demonstrating dependable navigation, object handling, and obstacle avoidance, the suggested robotic platform still has two major drawbacks for industrial deployment: a small payload capacity of 2 kg and a short operational endurance of 2–3 h. Potential enhancement pathways must be extensively assessed to guarantee scalability and practical use in warehouse settings. The relative advantages, drawbacks, viability, and anticipated effects of potential tactics are compiled in Table 4.

**TABLE 4:** Analysis of enhancement pathways for improving payload and endurance, highlighting strengths, limitations, feasibility, and expected impact.

Enhancement	Key strength	Limitation	Feasibility	Expected impact
Structural and actuator Upgrade	Increases payload capacity through stronger chassis and high torque lifting motors	Adds weight and raises energy demand; stability must be managed	Moderate – requires mechanical redesign	Extends payload range from 2 kg to 5–10 kg
High-capacity or hot-swappable batteries	Extends runtime by several hours or enables near-continuous operation	Heavier packs increase load; hot swapping requires safe connectors	High–mature technology with commercial availability	Improves endurance by 2–6 h, supporting full shifts
Automated docking and charging	Provides unattended recharging for continuous operation	Needs dedicated docking infrastructure and precise localization	Medium – costlier but scalable for multi-robot fleets	Enables sustained multi-shift deployment
Software-level power optimization	Reduces energy drawing via BMS integration and model compression	May slightly affect inference accuracy or processing speed	High – software-based and cost-effective	Boosts endurance by 10%–30% without hardware changes

This analysis emphasizes that to increase payload capacity, hardware-oriented improvements like structural reinforcement and actuator replacement are required, but they must be carefully weighed against stability and energy consumption. The most feasible short-term answer for endurance is offered by battery-related improvements, particularly hot-swappable designs, while automated docking and charging become increasingly important in large-scale deployments. Software-level power optimization, on the other hand, provides a quick, inexpensive way to increase runtime with little modification. When combined, these tactics provide a methodical road map for transforming the prototype into a reliable, expandable system fit for actual warehouse operations.

#### 4.5.1 Multi-robot scalability framework

**FIGURE 15:**
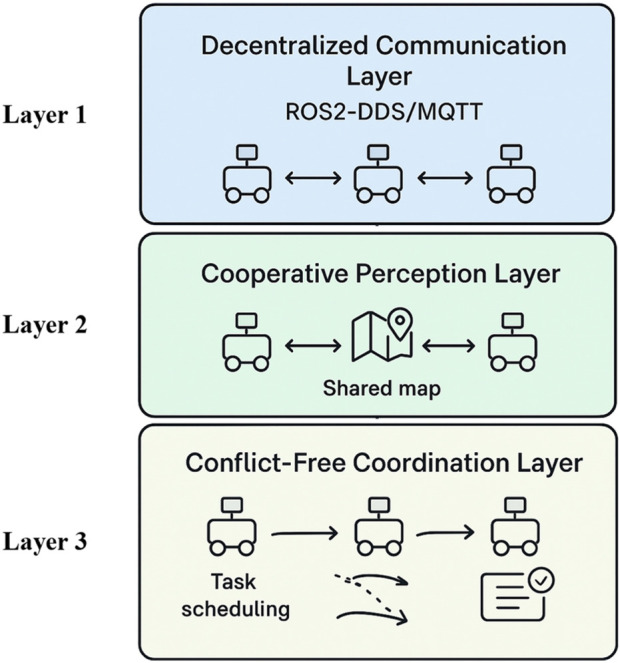
Three-layer scalability strategy for multi-robot systems containing decentralized communication, cooperative perception, and conflict-free coordination for safe and efficient warehouse operation.

To extend the current system toward large-scale deployments, a multi-robot scalability framework can be built on three strategic layers, as shown in Figure 15.

i. **Distributed Communication (Layer 1):** Every robot will function in a peer-to-peer communication system, such as lightweight MQTT or ROS2-DDS. By enabling direct communication between robots on task progress, navigation updates, and state information, this method does away with the need for a central controller and enhances resilience and fault tolerance in dynamic environments.
ii. **Shared Perception and Information Flow (Layer 2):** Robots can use edge-to-edge networks to send compressed sensory data, like obstacle detection and spatial mapping, to improve collective awareness. This maintains bandwidth efficiency while guaranteeing that perception transcends individual sensing limitations.
iii. **Coordinated Planning and Conflict Management (Layer 3):** A hybrid model can be used to control motion planning and task distribution. To maximise task assignment, reinforcement learning-driven scheduling will be integrated with priority-based decision procedures. Dynamic job distribution will be supported by auction-style allocation techniques, and navigation collisions will be less likely using predictive trajectory replanning.

By combining these layers, the framework facilitates safe navigation, effective data processing, and strong coordination, opening the door to scalable and dependable multi-robot systems that meet the requirements of Industry 4.0 logistical operations.

#### 4.5.2 User-centric evaluation and SME integration strategy

**FIGURE 16:**
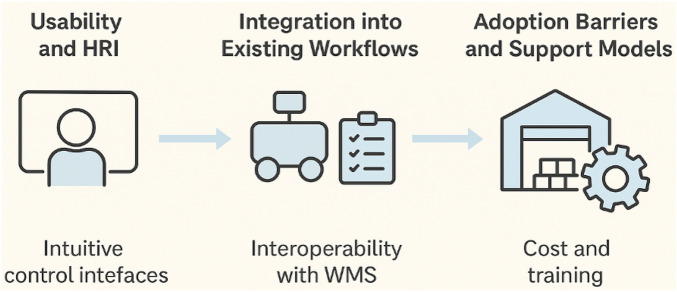
User-centric evaluation framework highlighting three focus areas for SME adoption: usability and human–robot interaction, integration with existing workflows, and addressing adoption barriers through cost-effective support models.

An additional crucial strategy is to investigate how user-related elements affect small and medium-sized businesses’ (SMEs) ability to successfully implement autonomous logistics robots. In contrast to major business entities with dedicated automation teams, SMEs frequently lack substantial technological know-how, which can lead to difficulties with system setup, implementation, and ongoing maintenance. As illustrated in Figure 16, there will be three main areas of focus for the research:

i. **Human–Robot Interaction (HRI) and usability:** Creating simple dashboards, user-friendly interfaces, and workflows requiring little setup so that operators without extensive training may efficiently monitor and manage robotic systems.
ii. **Integration into Existing Workflows:** To minimise interruption and lower training needs, research interoperability with current material flow and warehouse management system (WMS) systems.
iii. **Support Mechanisms and Adoption Barriers:** Reducing reliance on qualified personnel by offering lightweight solutions like plug-and-play modules, mobile-based control systems, and remote diagnostics to address SME-specific limitations about cost, training, and technical support.

The platform can be better tailored to the realities of SME operations by methodically resolving these organisational and human-centric issues. This will facilitate adoption and guarantee the platform’s long-term sustainability within Industry 4.0 logistics ecosystems.

## 5 Conclusion and future scope

This work offers a small, self-governing logistics robot that combines object handling, real-time navigation, and edge-based artificial intelligence perception on a single platform. The robot simultaneously performs path tracking, obstacle avoidance, and payload manipulation without relying on the cloud thanks to a customized ResNet-18 model installed on the NVIDIA Jetson Nano. With experimental validation in controlled and semi-structured environments, the system’s dependability for small- and medium-scale warehouse automation was confirmed with path accuracy of 92%, obstacle avoidance success of 88%, and object handling effectiveness of 90%. It provides a cost-effective substitute for conventional automation techniques that divide perception and actuation or depend on external processing because of its modular and energy-efficient architecture, which permits real-time autonomous operation.

Future work will strengthen safety-critical reliability by incorporating redundancy, adaptive re-planning, sensor fusion, and online learning mechanisms that enable real-time model updates during deployment. Stronger chassis components, better mechanical connections, and high-torque actuators can increase the payload capability beyond the existing 2 kg limits, guaranteeing steady and effective handling of heavier loads. To enhance autonomy in complex settings, reinforcement learning can be explored for navigation in unstructured environments, while visual SLAM can continue to support dynamic localization, and selective cloud-offloading to extend computational resources without compromising real-time performance. The framework can be expanded to facilitate multi-robot cooperation at the system level by means of fleet-level administration for effective and conflict-free operation, cooperative perception, decentralized communication, and coordinated task distribution. To ensure accessibility and useful adoption in industrial contexts with limited resources, a user-centric evaluation and SME-focused integration strategy can be adopted to provide lightweight support solutions, workflow compatibility, and easy operation.

## Data Availability

The raw data supporting the conclusions of this article will be made available by the authors, without undue reservation.
